# Neoadjuvant chemotherapy in breast cancer: a dose-dense schedule in real life and putative role of *PIK3CA* mutations

**DOI:** 10.18632/oncotarget.25270

**Published:** 2018-06-08

**Authors:** Valentina Cocciolone, Katia Cannita, Alessandra Tessitore, Valentina Mastroiaco, Lucia Rinaldi, Stefania Paradisi, Azzurra Irelli, Paola Lanfiuti Baldi, Tina Sidoni, Enrico Ricevuto, Antonella Dal Mas, Giuseppe Calvisi, Gino Coletti, Antonietta Ciccozzi, Laura Pizzorno, Valter Resta, Alberto Bafile, Edoardo Alesse, Corrado Ficorella

**Affiliations:** ^1^ Department of Biotechnological and Applied Clinical Sciences, University of L’Aquila, L’Aquila, Italy; ^2^ Medical Oncology Department, S. Salvatore Hospital, University of L’Aquila, L’Aquila, Italy; ^3^ Oncology Network ASL1 Abruzzo, UOSD Oncology Territorial Care, S. Salvatore Hospital, University of L’Aquila, L’Aquila, Italy; ^4^ Pathology Department, S. Salvatore Hospital, L’Aquila, L’Aquila, Italy; ^5^ Radiology Department, S. Salvatore Hospital, L’Aquila, L’Aquila, Italy; ^6^ Breast Unit, S. Salvatore Hospital, L’Aquila, L’Aquila, Italy

**Keywords:** neaodjuvant, dose-dense, PIK3CA, real life

## Abstract

**Background:**

Dose-dense chemotherapy is one of the treatments of choice for neoadjuvant therapy in breast cancer (BC). Activating mutations in *PIK3CA* gene predict worse response to neoadjuvant chemotherapy for HER2-positive patients, while their role is less clearly defined for HER2-negative tumors.

**Methods:**

We conducted a phase I/II study of neoadjuvant, sequential, dose-dense anthracycline/taxane chemotherapy, plus trastuzumab in HER2-positive patients and investigated the correlation of pre-treatment *PIK3CA* mutation status with pathologic complete response (pCR) and long-term outcome in a real-life setting.

**Results:**

we established a dose-dense docetaxel recommended dose of 60 mg/m^2^ and 65 mg/m^2^, with or without trastuzumab, respectively, according to HER2-status, following dose-dense epirubicin-cyclophosphamide (90/600 mg/m^2^), every 2 weeks. The overall pCR rate was 21.4%; median disease-free survival (DFS) was 52 months and median overall survival (OS) was not yet reached. *PIK3CA* mutation status was not significantly associated with the pCR rate: 18% for both mutated and wild-type patients. The pCR rate was: 25% in the mutated and 24% in the wild-type (p 0.560) cohort of the HER2-positive subgroup; 33% both in the mutant and wild-type cohort of the triple-negative subgroup; no pCR neither in the mutant nor in the wild-type cohort of the HR-positive/HER2-negative subgroup. Among the HER2-positive population, a trend toward worse DFS was observed in case of mutation, as opposed to the triple negative population.

**Conclusions:**

This study proposes an effective and safe neoadjuvant dose-dense anthracycline/taxane schedule and suggests that *PIK3CA* mutation analysis can be usefully performed in real-life clinical practice.

## INTRODUCTION

Neoadjuvant chemotherapy is considered the standard therapeutic approach to locally advanced breast cancer (LABC) and inflammatory breast cancer (IBC), but it should be considered the treatment of choice, in clinical practice, even in operable disease, based on the results of several randomized clinical trials showing its equivalence, in terms of DFS and OS, to adjuvant therapy [1]. Obtaining a pathologic complete response (pCR), defined as the absence of residual invasive carcinoma within both the breast and axillary lymph nodes after neoadjuvant treatment [2], has a significant impact on survival, enough to be considered a powerful surrogate marker [3–5]. Frequency of pCR in patients with low-grade, hormone receptor (HR)-positive tumors is low, and more than doubled in the high-grade HR-positive tumors. Triple negative and human epidermal growth factor receptor 2 (HER2)-positive tumors are more likely able to achieve a pCR; within the HER2-positive population, pCR is more common for HR-negative than for HR-positive tumors, and with the addition of trastuzumab. The most favorable outcomes after pCR are detected in patients with HER2-positive/HR-negative tumors who receive trastuzumab, and in the triple-negative subgroup [6]. The results of the B-27 trial showed an 87% and a 16% increase in pCR rate and in negative axillary nodes, respectively, with the sequential addition of preoperative docetaxel after completion of preoperative doxorubicin-cyclophosphamide. The addition of trastuzumab to neoadjuvant chemotherapy in the HER2-positive disease significantly increased the pCR rate compared to chemotherapy alone [7, 8], with a favorable impact on the event-free survival, strongly associated with pCR only in patients treated with trastuzumab [9]. Based on the Gompertzian kinetic model of cancer growth [10], increasing dose density improves outcomes [11] by killing more cancer cells as they re-grow after the previous cycle of therapy, making reasonable to hypothesize that shortening the interval between cycles of chemotherapy may be more critical in high-grade rapidly proliferating tumors [12]. A meta-analysis of randomized controlled trials, comparing dose-dense (dd) chemotherapy with a conventional schedule in non-metastatic BC, showed that the dose-dense approach results in better OS and DFS, particularly in women with HR-negative disease, even if it was associated with a greater incidence of non-hematological adverse events [13].

Therefore, we conducted a phase I/II study of neoadjuvant dd anthracycline/taxane chemotherapy (plus trastuzumab (T) in HER2-positive disease) in BC patients, planned to define the recommended dose (RD) of dd Docetaxel (D) following dd Epirubicin/Cyclophosphamide (EC).

In recent years, increasing importance is gaining the evaluation of the phosphatidylinositol-3-kinase (PI3K) mutational status as a prognostic and predictive factor. Mutations of the p110α catalytic domain of PI3K, encoded by the *PIK3CA* gene, are the most common genetic alterations of the PI3K/Akt/mammalian target of rapamycin (mTOR) pathway in BC, being identified in approximately 20% of all BCs. About 80% of them occur at the level of the α-helical (Exon 9) and kinase domain (Exon 20) of the p110α subunit, involving three hotspot sites [14]: a hotspot in the kinase domain, with the aminoacidic substitution of H1047R, inducing a conformational change in the protein activation loop, responsible for enzymatic hyperactivity; two other hotspots in the helical domain, consisting of the E542K and E545K substitutions, resulting in ineffective regulation of the p110α kinase activity, mediated by the regulatory subunit. In BC, mutations in Exon 20 are more frequent than in Exon 9 [15]. However, the frequency of *PIK3CA* mutations is not equally distributed among the different biologic subtypes [16]: a gene mutation has been reported up to 45% in Luminal-A BCs , in 29% of Luminal-B and in 39% of HER2-positive tumors, while approximately 9% of basal-like tumors harbor a *PIK3CA* mutation.

In the neoadjuvant setting, *PIK3CA* mutations do not appear to be associated with altered sensitivity to preoperative chemotherapy with anthracyclines and taxanes, although mutations in exon 9 are associated with a higher rate of node-negative residual disease, especially in the subgroup of patients with estrogen receptor (ER)-positive BC [17]. Analyses from patients with HER2+ BC treated with neoadjuvant anthracyclines, taxanes and anti-HER2 agents, showed that activating mutations in *PIK3CA* predict a lower chance of achieving a pCR, while they did not significantly impact DFS and OS [18, 19]. The negative prognostic impact of *PIK3CA* mutations in BC is more evident in the metastatic disease: worse median progression-free survival was observed for patients whose tumors expressed mutated versus wild-type *PIK3CA*, in both the control (8.6 versus 13.8 months) and pertuzumab groups (12.5 versus 21.8 months) [20].

In the light of these premises, in this study we also assessed the correlation of pre-treatment *PIK3CA* mutation status with pCR, relapse-free survival (RFS), DFS and OS of enrolled patients, according to the histological subgroups.

## RESULTS

### Baseline characteristics

From October 2010 to August 2015, 42 consecutive, unselected patients were enrolled (Table 1), 12 in the phase I part of the study. Median age was 50 years (range 38-66). As to disease extension, 19 patients (45.5%) had operable stage II-IIIA BC, 22 (52.5%) LABC and 1 patient (2%) IBC. Out of 44 evaluable tumors (2 patients had bilateral carcinoma), 54.5% were HER2-positive (ER+/Progesterone Receptor (PgR)+, 16%; ER+/PgR-, 13.5%; ER-/PgR-, 25%) and 45.5% HER2-negative (ER+/PgR+, 18.5%; ER+/PgR-, 13.5%; ER-/PgR-, 13.5%).

**Table 1:** Patients characteristics

	N. (%)
**N. patients**	42
**Age, years median**	50
range	38-66
**Menopause Status**	
Pre	28 (67)
Post	14 (33)
**WHO Performance Status**	
0	40 (95)
1	2 (5)
**CIRS**	
Primary	27 (64)
Intermediate	12 (29)
Secondary	3 (7)
**Histological type**	
Ductal	36 (86)^*^
Lobular	5 (12)^*^
Other ^*^(2 pts with bilateral breast cancer)	3 (7)
**Stage of disease**	
Operable EBC	19 (45.5)
LABC	22 (52.5)
IBC	1 (2)
**Cardiovascular comorbidity**	
Yes	9 (21)
No	33 (79)

### Dose-finding

Overall, 5 dose-limiting toxicities (DLTs) were observed out of 12 patients (42%). Among HER2-positive patients, DLTs occurred in all 3 patients enrolled in the first cohort at D 65 mg/m^2^, represented by G3 asthenia in the first 2 patients, occurred at the second and fourth cycle, respectively, and G2 asthenia lasting more than 2 weeks in the third patient, occurred at the first cycle. Therefore, a next cohort of 3 patients was necessarily enrolled at D 60 mg/m^2^; at this dose-level, no DLT was reported. Thus, 60 mg/m^2^ was established as D RD in combination with T. Among HER2-negative patients, DLT, represented by G3 hand-foot syndrome (HFS), occurred in 1 out of 3 patients enrolled in the first cohort at D 65 mg/m^2^, so a new cohort was enrolled at the same dose-level and 1 DLT, represented by G2 anemia for more than 2 weeks, occurred. Thus, 65 mg/m^2^ was D RD when administered alone.

### Treatment administration

Out of 42 enrolled pts, 38 (90%) completed the treatment cycles planned in the study (4 cycles of EC and 5-6 cycles of D with/without T), as two patients, 1 of the HER2-positive and 1 of the HER2-negative group, reported a hypersensitivity reaction to D at the second administration and continued treatment with nanoparticle albumin-bound (nab)-Paclitaxel and two patients, in the HER2-negative subgroup, refused to undergo the last D cycle to anticipate surgery. The two patients who had the hypersensitivity reaction to D were not included in the D safety analysis. The total number of administered cycles was 395 (168 cycles of EC, 128 cycles of D with T and 99 cycles of D alone). Overall, median absolute dose intensity (DI) of E was 45 mg/m^2^/week (range 33.3-45); median absolute DI of D in HER2-negative and HER2-positive population was 30.5 mg/m^2^/week (range 23.5-32.5) and 29.75 mg/m^2^/week (range 24.2-30), respectively.

### Activity and efficacy

Activity and efficacy data are shown in Table 2. In the intention-to-treat (ITT) analysis, all 42 enrolled patients were evaluable for activity: pCR was obtained in 8 patients (19%). In the as-treated analysis, 40 patients were evaluable, as two patients reported a hypersensitivity reaction to D at the second administration and continued treatment with nab-Paclitaxel: pCR was achieved in 8 patients (20%). As one of the two patients treated with nab-Paclitaxel obtained a pCR, the pCR rate in the whole population treated with sequential EC and taxanes was 21.4% (9 out of 42 patients). Four of these pCRs were characterized by residual carcinoma *in situ* in the breast with negative nodes. As two patients had a synchronous bilateral BC, the subgroup activity analysis was performed on 42 tumors treated with EC → D ± T and 44 tumors treated with EC → Taxane ± T. In the as-treated analysis (42 tumors), in the HER2-positive population, treated with D in combination with T, pCR was observed in 6 out of 23 tumors (26%); 3 pCRs were reported out of 13 ER-positive tumors (23%) and 3 out of 10 ER-negative tumors (30%). In the HER2-negative population, pCR was observed in 3 out of 19 tumors (15.7%); 2 pCRs were reported out of 6 triple-negative tumors (TNBC) (33.3%) and 1 out of 13 ER-positive/HER2-negative tumors (7.7%) (Table 3a). As the patient treated with nab-Paclitaxel who reported the pCR had a HR-negative/HER2-positive tumor, in the ITT analysis of patients treated with EC → Taxane ± T (44 tumors), pCR was observed in 7 out of 24 tumors (29.2%) in the HER2-positive population: 3 pCRs were reported out of 13 ER-positive tumors (23%) and 4 out of 11 ER-negative tumors (36.4%). In the HER2-negative population, pCR was observed in 3 out of 20 tumors (15%); 2 pCRs were reported out of 6 triple-negative tumors (33.3%) and 1 out of 14 ER-positive/HER2-negative tumors (7.1%) (Table 3b). After a median follow-up of 33 months for both ITT and as-treated analysis, overall median RFS and DFS were 57 months and 52 months, respectively: 16 patients underwent distant disease recurrence (5 on brain, 2 on liver, 1 on lung and pleura and 4 on lymph nodes) and 2 patients had a local recurrence on the breast (including cutaneous diffusion and regional lymph nodes involvement); 1 patient had a second tumor on esophagus. Median RFS was not reached, 57 months and 12 months for the HER2+, HR+/HER2- and triple-negative subgroup, respectively; median DFS was not reached, 52 months and 7.5 months for the HER2+, HR+/HER2- and triple-negative subgroup, respectively (Table 4). Out of 23 HER2-positive patients, 7 (30%) disease recurrences occurred [3 out of 11 ER-/PgR-/HER2+ (27%) and 4 out of 12 ER+/PgR+ or -/HER2+ (33%)]. Out of 6 triple-negative patients, 4 (67%) disease recurrences occurred, and one patient had the esophageal tumor. Out of 13 ER+/PgR+ or -/HER2-negative patients, 5 (38%) disease recurrences occurred [3 out of 7 ER+/PgR+/HER2- (43%), and 2 out of 6 ER+/PgR-/HER2- (33%)]. Overall median OS was not reached: 10 patients died due to BC, 1 patient died due to esophageal tumor. Median OS was not reached for the HER2+ and HR+/HER2- subgroup and 29.5 months for the triple-negative subgroup. Ten patients died due to BC: HER2-positive, 3; triple-negative, 4; HR+/HER2-negative, 3; 1 patient with TNBC died due to esophageal tumor.

**Table 2:** Activity and efficacy

		Intention-to-treat analysis		As-treated analysis	
		N.	%	N.	%
**ddEC → D ± T**	**Enrolled patients**	42	100	42	100
	**Evaluable patients**	42	100	40	95
	**pCR**	8	19	8	20
**ddEC → Taxane ± T**	**Enrolled patients**	42	100	42	100
	**Evaluable patients**	42	100	42	100
	**pCR**	9	21.4	9	21.4
**Median RFS, months**		57 mo			
Range		7-65+			
Progression events		16 (+1)^*^			
**Median DFS, months**		52 mo			
Range		2-58+			
Progression events		16 (+1)^*^			
**Median OS, months**		nr			
Range		9-71+			
Deaths		10 (+1)^*^			

^*^1 patient had a second tumor on esophagus and died because of it.

**Table 3A:** pCR according to BC subtype on as-treated 42 evaluable tumors

		HER2	
		positive	Negative
		D+T	D alone
	**negative**	3/10 (30%)	2/6 (33.3%)
**ER**	**positive**	3/13 (23%)	1/13 (7.7%)
	**Overall**	6/23 (26%)	3/19 (15.7%)

**Table 3B:** pCR according to BC subtype on ITT 44 evaluable tumors

		HER2	
		positive	Negative
		D+T	D alone
	**negative**	4/11 (36.4%)	2/6 (33.3%)
**ER**	**positive**	3/13 (23%)	1/14 (7.1%)
	**Overall**	7/24 (29.2%)	3/20 (15%)

**Table 4:** Efficacy according to subtypes

	HER2 +	HR+/HER2 -	TNBC
**Median RFS, months**	nr	57 mo	12 mo
Progression events	7	5	4 (+1)^*^
**Median DFS, months**	nr	52 mo	7.5 mo
Progression events	7	5	4 (+1)^*^
**Median OS, months**	nr	nr	29.5 mo
Deaths	3	3	4 (+1)^*^

^*^1 patient had a second tumour on esophagus and died because of it.

### Toxicity

All patients were evaluated for EC toxicity, 22 patients for D in combination with T and 18 for D alone toxicity. During the EC phase, regarding non-hematological toxicity, asthenia was the most common severe toxicity (7% of patients, 4% of cycles). The incidence of other G3 non-hematological toxicities was rare, and included only vomiting and transaminases increase in 2% of patients, 0.5% of cycles. The single severe hematological toxicity was neutropenia (with or without leucopenia), which occurred in 40% of patients (14% G3 and 26% G4) and 15% of cycles. Febrile neutropenia was not reported (Supplementary Table 1). During the D phase, a distinction must be done between the HER2-positive group, treated with D 60 mg/m^2^ in combination with T, and the HER2-negative group, treated with D 65 mg/m^2^ alone. Regarding non-hematological toxicity, asthenia was once again the most frequent toxicity, occurring at G3 in 18% of patients and 3% of cycles, and in 11% of patients and 2% of cycles in the HER2-positive and HER2-negative group, respectively. Anyway, G2 asthenia was more frequent in the HER2-negative group (56% versus 3% of patients). Severe transaminases increase was reported by 1 patient (5%) in 1 cycle (0.7%) in the HER2-positive group, while severe myalgia occurred only in the HER2-negative group, by 1 patient (6%) in 1 cycle (2%). As to hematological toxicity, G3-4 neutropenia (with or without leucopenia) occurred in 12% of patients (6% G3 and 6% G4) in 3% of cycles, only in the D alone arm (Supplementary Table 2). Cumulatively, cardiac toxicity was reported in 28 out of all 42 enrolled patients (67%) and 39 cycles (10%), with no limiting events, thus represented only by cardiac dysfunctions, not requiring treatment delay or interruption (Supplementary Table 3a). Neither c-TnI nor precursor-Brain-Natriuretic-Peptide (pBNP) increase was reported. Two patients, one with HER2-positive and one with HER2-ngative tumor, had an asymptomatic ≤10% left ventricular ejection fraction (LVEF) reduction from baseline with a final value, at treatment completion, of 50%. The mean overall LVEF baseline value was 65%, maintained after the first treatment phase with EC (64%) and up to chemotherapy completion (65%). In the HER2-positive population, median LVEF was 65%, 64% and 61% at baseline, after EC and at treatment completion, respectively; in the HER2-negative population, median LVEF was maintained at 65% in all three evaluations (Supplementary Table 3b).

### Frequency of *PIK3CA* mutation and association with clinicopathological factors

**Figure 1:**
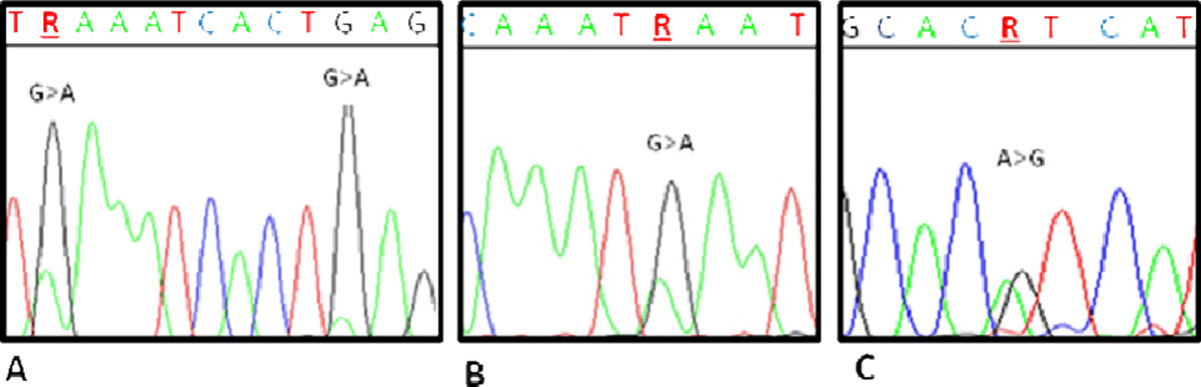
Electropherograms showing *PIK3CA* mutations **(A)** Sample AQ03: *PIK3CA* exon 9 c.1624G>A, E542K; c.1633G>A, E545K. **(B)** Sample AQ02: *PIK3CA* exon 20 c.3129G>A, M1043I. **(C)** Sample AQ08: *PIK3CA* exon 20 c.3140A>G, H1047R.

Thirty-nine out of 42 tumor samples (92.8%) were analyzed for exon 9 and 20 *PI3KCA* mutations. Mutations of exon 9 were more common (9/39, 23%) than mutations of exon 20 (1/39, 3%); one of the tumors (3%) harbored a mutation in both exons. Mutations detected in exon 9 were c.1624G>A (corresponding to the E542K amino acid substitution) alone in 8 cases, associated with c.1633G>A (corresponding to the E545K amino acid substitution) in 2 cases; mutations detected in exon 20 were c.3140A>G (corresponding to the H1047R amino acid substitution) in 1 case and c.3129G>A (corresponding to the M1043I amino acid substitution) in 1 case (Table 5) (Figure 1). The latter mutation was described as very infrequent in the Cosmic database ( http://cancer.sanger.ac.uk/). No significant correlation between *PIK3CA* mutation status and other baseline characteristics was observed. A *PIK3CA* mutation was detected in 4/21 (19%) HER2-positive tumors, 3/6 (50%) triple-negative tumors and 4/12 (33%) HR-positive/HER2-negative tumors, with not statistically significant differences between subgroups (p 0.296).

**Table 5:** Characteristic*s* of patients carrying *PIK3CA* mutations

	RE/RPg	HER2	Activity	DFS	RFS	OS	*PIK3CA* ex9	*PIK3CA* ex20
**HER2-positive**								
AQ01	95/40	pos	ypT2 ypN3a	11	17	25+	c.1624G>A	wt
AQ02	95/95	pos	ypT1b ypN0	21+	27+	27+	wt	c.3129G>A
AQ03	neg/neg	pos	pCR	35+	41+	41+	c.1624G>A c.1633G>A	wt
AQ04	neg/neg	pos	ypT1c ypN2	2	7	43+	c.1624G>A	wt
**TNBC**								
AQ05	neg/neg	neg	pCR	29+	36+	36+	c.1624G>A c.1633G>A	wt
AQ06	neg/neg	neg	ypT1c ypN0	26	31	33	c.1624G>A	wt
AQ07	neg/neg	neg	ypT1a ypN0	6	10	26	c.1624G>A	wt
**HR+/HER2-**								
AQ08	30/neg	neg	ypTx ypN1	47+	54+	54+	c.1624G>A	c.3140A>G
AQ09	65/5	neg	ypT1c ypN0	28+	33+	33+	c.1624G>A	wt
AQ10	80/1	neg	ypT3 ypN3	3	7	19	c.1624G>A	wt
AQ11	70/60	neg	ypT1a ypN0	60+	65+	65+	c.1624G>A	wt

c.1624G>A corresponds to E542K; c.1633G>A corresponds to E545K; c.3140A>G corresponds to H1047R; c.3129G>A corresponds to M1043I.

### Association of *PIK3CA* mutations and response to treatment

The presence of a *PIK3CA* mutation, overall, was not significantly associated with the pCR rate: 2 out of 11 (18%) patients with *PIK3CA*-mutated tumors achieved a pCR compared to 5 out of 28 (18%) patients in the wild-type cohort (odds ratio (OR), 1.022; 95% CI, 0.167 to 6.261; p 0.981). Of note, the tumor tissue of 2 patients achieving pCR was not available for *PIK3CA* mutational analysis. By subgroups, in the HER2-positive population, the pCR rate was 25% (1/4) in the mutated cohort and 23.5% (4/17) in the wild-type cohort (OR, 1.083; 95% CI, 0.087 to 13.55; p 0.950); in the triple-negative population, it was 33% both in the mutated (1/3) and in the wild-type cohort (1/3) (OR, 1.000; 95% CI, 0.033 to 29.83; p 1.000); in the HR-positive/HER2-negative population, no pCR was obtained neither in the mutated (0/4) nor in the wild-type (0/8) cohort. Within the HER2-positive subgroup, the pCR rate was 33% (3/9) for the HR-negative and *PIK3CA* wild-type cohort and 50% (1/2) for the HR-negative and *PIK3CA* mutated cohort (OR, 0.5; 95% CI, 0.023 to 11.10; p 0.658); 12.5% (1/8) for the HR-positive and *PIK3CA* wild-type cohort, while any patient in the HR-positive and *PIK3CA* mutated cohort achieved the pCR (OR, 1.000; 95% CI, 0.030 to 33.35; p 0.598). The response to treatment according to the Residual Cancer Burden (RCB) score was not significantly related to the *PIK3CA* mutational status, as the frequency of *PIK3CA* mutations was similar in patients with pCR, RCB-I, RCB-II or RCB-III (p 0.454) (Table 6).

**Table 6:** Association of *PIK3CA* genotype and response to treatment

		*PIK3CA* mutated N (%)	*PIK3CA* wild-type N (%)	p value^a^
pCR versus Residual Disease (nv → 3 patients)				0.981
	RD	9/11 (82)	23/28 (82)	
	pCR	2/11 (18)	5/28 (18)	
Residual Cancer Burden (nv → 8 patients)				0.454
	0	2/10 (20)	5/24 (21)	
	I	1/10 (10)	2/24 (8)	
	II	5/10 (50)	6/24 (25)	
	III	2/10 (20)	11/24 (46)	
pCR				
HER2+				
	HR+	0/2	1/8 (12.5%)	0.598
	HR-	1/2 (50%)	3/9 (33%)	0.658
	Total	1/4 (25%)	4/17 (23.5%)	0.950
HER2-				
	HR+	0/4	0/8	nv
	HR-	1/3 (33%)	1/3 (33%)	1.000
	Total	1/7 (14%)	1/11 (9%)	0.732

^a^ Chi-square test.

### Association between *PIK3CA* genotype and long-term survival

**Figure 2:**
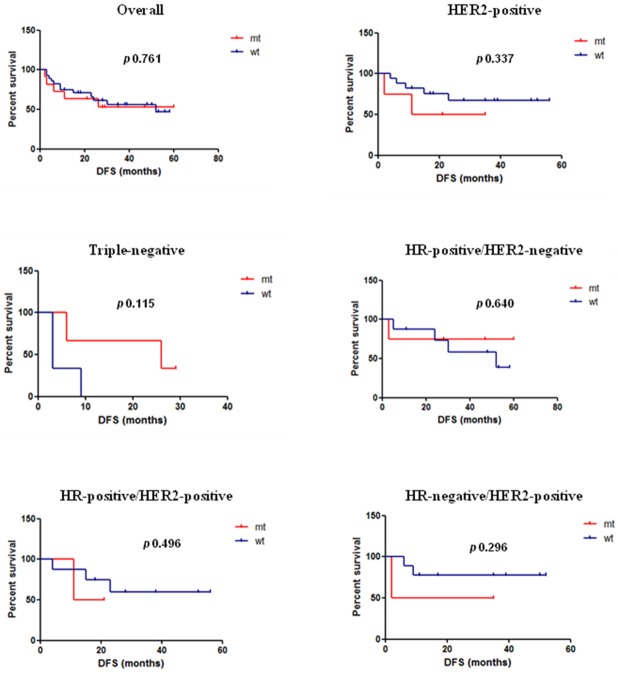
Kaplan–Meier curves of DFS overall and by subgroups according to *PIK3CA* genotype Median DFS overall and by subgroups in the mutated versus wild-type cohort, respectively. Overall: not reached versus 52 months (p 0.761); HER2-positive: 23 months versus not reached (p 0.337); triple-negative: 26 months versus 3 months (p 0.115); HR-positive/HER2-negative: not reached versus 52 months (p 0.640); HR-positive/HER2-positive: 16 months versus not reached (p 0.496); HR-negative/HER2-positive: 18.5 months versus not reached (p 0.296).

Overall, neither DFS nor OS were statistically significantly different between patients with or without a *PIK3CA* mutation: median DFS was not reached in the mutated cohort versus 52 months in the wild-type cohort (hazard ratio, 1.185; 95% CI, 0.396 to 3.541; p 0.761); median OS was not reached in both the mutated and wild-type cohort (hazard ratio, 0.933; 95% CI, 0.250 to 3.478; p 0.918). Within the HER2-positive subgroup, median DFS was 23 months in the mutated cohort versus not reached in the wild-type cohort (hazard ratio, 2.731; 95% CI, 0.350 to 21.28; p 0.337); median OS was not reached in both the mutated and wild-type cohort (hazard ratio, 0.285; 95% CI, 0.016 to 4.940; p 0.389). In this subgroup, similar results were obtained when analyzing data based on HR status: within the HR-positive/HER2-positive population, median DFS was 16 months in the mutated cohort versus not reached in the wild-type cohort (hazard ratio, 2.784; 95% CI, 0.146 to 53.05; p 0.496); median OS was not reached in both the mutated and wild-type cohort (hazard ratio, 0.286; 95% CI, 0.0021 to 38.47; p 0.617); within the HR-negative/HER2-positive population, median DFS was 18.5 months in the mutated cohort versus not reached in the wild-type cohort (hazard ratio, 6.041; 95% CI, 0.207 to 176.3; p 0.296); median OS was not reached in both the mutated and wild-type cohort (hazard ratio, 0.263; 95% CI, 0.0028 to 24.36; p 0.563). Differences in outcome between HR-negative/HER2-positive mutated cohort and HR-positive/HER2-positive mutated cohort were not significantly different (DFS: hazard ratio, 1.423; 95% CI, 0.082 to 24.66; p 0.808. OS: any event of death). Within the triple-negative subgroup, median DFS was 26 months versus 3 months in the mutated and wild-type cohort, respectively (hazard ratio, 1.173; 95% CI, 0.019 to 1.53; p 0.115); median OS was 33 months versus 19 months in the mutated and wild-type cohort, respectively (hazard ratio, 0.563; 95% CI, 0.071 to 4.446; p 0.586). Within the HR-positive/HER2-negative subgroup, median DFS was not reached in the mutated cohort versus 52 months in the wild-type cohort (hazard ratio, 0.631; 95% CI, 0.091 to 4.34; p 0.640); median OS was not reached in both the mutated and wild-type cohort (hazard ratio, 1.005; 95% CI, 0.09 to 11.14; p 0.997) (Table 7). Differences in outcome between HR-positive/HER2-negative mutated cohort and HR-positive/HER2-positive mutated cohort were not significantly different (DFS: hazard ratio, 0.561; 95% CI, 0.031 to 10.03; p 0.694; OS: hazard ratio, 4.482; 95% CI, 0.070 to 286.5; p 0.479). Kaplan–Meier curves of DFS are shown in Figure 2.

**Table 7:** Association between *PIK3CA* genotype and long-term survival

	Disease-Free Survival (months and events/patients)		p value^a^
	*PIK3CA* mutated	*PIK3CA* wild-type	
Overall	**nr** (4/11)	**52** (12/28)	0.761
HER2+			
Overall	**23** (2/4)	**nr** (5/17)	0.337
HR+	**16** (1/2)	**nr** (3/8)	0.496
HR-	**18.5** (1/2)	**nr** (2/9)	0.296
HR+/HER2-	**nr** (1/4)	**52** (4/8)	0.640
TNBC	**26** (1/3)	**3** (3/3)	0.115

^a^ Chi-square test.

## DISCUSSION

The present phase I-II study proposes a sequential regimen of dose-dense EC followed by dose-dense D with or without T, according to HER2-status, at D RD of 60 mg/m^2^ and 65 mg/m^2^, respectively, over a 20-weeks period before surgery. The dose-dense approach allows to safely administer D at a median DI of 29.75 mg/m^2^/week in the HER2-positive population and 30.5 mg/m^2^/week in the HER2-negative one, by improving the toxicity profile. The maintenance of an adequate DI was the limit of several studies testing, in the neoadjuvant setting, the sequence or combination of anthracyclines and taxanes in a dose-dense schedule: they could not demonstrate the feasibility, in clinical practice, of D at 100 mg/m^2^ every 2 weeks [21, 22], except when planning an inversion of the sequence of drugs, that is administering taxanes before the anthracycline [23].

As we conducted a monocentric study, achieving the sample size planned to complete the enrollment required for the phase II, takes too much time. Therefore, the enrollment has been closed in advance. Anyway, in the phase I of the study we identified the recommended doses of docetaxel in the two different subgroups of patients and the preliminary data of the phase II analysis show that the proposed regimen produces results that are consistent with those of the major and larger studies of sequential anthracyclines and taxanes in alternative schedules. In fact, the overall 19% pCR rate obtained in the present study with EC → D and 21.4% in the overall study with sequential anthracycline and taxane, is comparable to 17.1% achieved by the sequential regimen dd EC followed by D plus capecitabine we previously reported [24] and to those achieved in the largest 3-weekly anthracyclines-taxanes sequential trials. In the NSABP B-27, pCR on breast and axillary nodes (absence of invasive cancer cells) was obtained in 21.8% of patients treated with 4 cycles of AC (60/600 mg/m^2^) every 21 days, followed by 4 cycles of D (100 mg/m^2^) every 21 days (group II). Anyway, the cumulative treatment completion rate was 80.7% (78.8% in group II) and 10.9% of patients discontinued D because of adverse events [25]. In the Her2-positive subgroup, we obtained a pCR rate of 26% if considering EC → D and 29.2% if considering the overall population treated with sequential anthracycline and taxane. Even if this pCR rate is lower than the 38% expected according to the meta-analysis by Valachis et al [26], it should be noted that it included studies where trastuzumab was administered upfront, concomitantly with both anthracyclines and taxanes, where efficacy seemed to be better compared to studies not combining anthracyclines and trastuzumab. Anyway, in the study by Pierga et al [27], where patients received EC (75/750 mg/m^2^) every 3 weeks for 4 cycles followed by D (100 mg/m^2^) every 3 weeks for 4 cycles with trastuzumab, 26% had a pCR (no residual invasive cells on breast and nodes), with a good toxicity profile and no cardiac event. Our pCR rate, comparable to this, was obtained in a shorter period, and with any toxicity increase. The higher pCR rate in the HR-/HER2+ subgroup, compared to the HR+/HER2+ subgroup (30% vs 23% with EC → D and 36.4% vs 25% with sequential anthracycline and taxane), is in line with literature data. With the 3-weekly schedule just mentioned, 32% of patients in the ER-/PgR- group and 20.5% in the ER+ and/or PgR+ group achieved a pCR [27]; in the TECHNO study, the pCR rates were 42.3% and 35.4% for patients in the HR- and HR+ populations, respectively [28]; Buzdar al reported a 70.4% of pCR rate in women with ER-/PgR- BC versus 47.6% in women with ER+ or PgR+ disease, with trastuzumab administered with paclitaxel following FEC [29]. Since, so far, there are no studies investigating D in combination with trastuzumab in a dose-dense schedule, we cannot know how much our regimen could be improved, unless hypothesizing the introduction of a dual HER2-blockade, that showed high efficacy compared to trastuzumab alone [30–34]. In the triple-negative population, we obtained a pCR rate of 33.3%, comparable to those obtained, in this subset, with sequential anthracyclines-taxanes regimens [25, 27, 35, 36] and to the meta-analytic results by Houssami et al [37]. There are potential reasons to believe that the benefits of dose-dense therapy could be particularly effective for specific subtypes such as triple-negative tumors, biologically characterized by a high proliferation rate and constitutive expression of genes related to proliferation [38]. On the other hand, TNBC is a highly diverse group of cancers and further sub-classification is needed for predicting the benefits of chemotherapy [39]. The challenge is therefore to identify subsets of patients with high probability of achieving a pCR, allowing them to have a good long-term prognosis after neoadjuvant chemotherapy, considering the high relapse rate among TNBC patients with residual disease [40]. In the HR-positive/HER2-negative population, we obtained a pCR rate of 7.7% if considering EC → D and 7.1% if considering the sequential anthracycline and taxane chemotherapy. The <10% pCR rate in this subgroup, comparable with a meta-analytic pCR rate of 8.3% [37], imposes a better selection of patients, proposing neoadjuvant chemotherapy only to those with inoperable disease or candidate to adjuvant chemotherapy, for example for clinical node positive status, absent or low expression of PgR or high Ki67, which seem to influence the response to neoadjuvant treatment within the group of luminal tumors [41, 42]. As to disease recurrences, we observed a higher rate of incidence in the triple-negative subgroup (67%), which is the only one where median RFS, DFS and OS were reached: 12 months, 7.5 months and 29.5 months, respectively. Notably, among patients with TNBC, recurrences occurred even if ypN0 at surgery, as if nodal status at surgery did not influence long-term outcome of patients with this biologically aggressive disease. Conversely, among patients with HR+/HER2- tumors, recurrences occurred only in ypN+ patients at surgery, confirming the negative prognostic impact on survival of nodal residual disease after neoadjuvant chemotherapy [43].

In the present study, *PIK3CA* mutational analysis did not define subgroups of patients most likely to achieve the pCR. Within the HER2-positive tumors, the prevalence of *PIK3CA* mutations (19%) is comparable to results from neoadjuvant trials in the HER2-positive BC population, ranging from 19.2% to 24.3% [18, 19, 34]. In this subgroup, we obtained a pCR rate of 25% in the mutated cohort and 23.5% in the wild-type. A pooled analysis of 967 patients from five prospective trials investigating double blockade with lapatinib and trastuzumab in the neoadjuvant setting, recently demonstrated that *PIK3CA* mutations negatively influence pCR: overall, HER2-positive tumors harboring mutations had significantly lower pCR rates compared to wild-type (16.2% versus 29.6%; p <0.001). Anyway, a significantly different pCR rate between *PIK3CA* mutated and wild-type tumors could only be observed in the group receiving trastuzumab and lapatinib (16.7% versus 39.1%; p <0.001), while only a trend toward worse response was observed in the cohort of patients treated with trastuzumab alone, whose pCR rate was 20.3% versus 27.1% for the mutated and wild-type tumors, respectively (p 0.343) [44]. Consistently with our findings, an apparently lower pCR rate for *PIK3CA* wild-type compared to mutated patients, in individuals treated with chemotherapy plus trastuzumab alone, was observed in the CHER-LOB study [45]. In this regard, a recent retrospective integrated analysis on HER2-positive patients treated with anthracycline-taxane-based chemotherapy plus trastuzumab as single blockade within the GeparQuattro study, PTEN and pEBP4, a PI3K downstream marker, added information to the predictive role of *PIK3CA* mutation status [46]. In line with results from the mentioned pooled analysis [44], an interaction between *PIK3CA* and HR status can be identified also in our analysis, even if not significant. HR-positive and *PIK3CA* mutated patients have the lowest incidence of pCR (any patient in this group achieved it), compared to HR-positive and *PIK3CA* wild-type (12.5%), HR-negative and *PIK3CA* mutated (50%) and HR-negative and *PIK3CA* wild-type (33%) patients. Among patients with triple-negative and HR-positive/HER2-negative tumors, *PIK3CA* mutational status did not affect the pCR rate. In the triple-negative subgroup, the pCR rate was 33% both for the mutated and the wild-type tumors, while in the HR-positive/HER2-negative subgroup any patients achieved pCR, neither wild-type nor mutated. To the best of our knowledge, to date no clinical studies have been performed to evaluate the role of *PIK3CA* mutations in the achievement of the pCR in these subgroups of patients. The activation of the PI3K pathway has been identified as an important issue in triple-negative/basal-like BC. As recently demonstrated, *PIK3CA* kinase domain mutations can be frequently detected in androgen receptor (AR)-positive TNBC compared to the other subtypes (40% versus 4%) and targeting AR in preclinical study increases sensitivity to PI3K inhibitors [47]. An open label phase II randomized study, evaluating the addition of the mTOR inhibitor everolimus to a standard neoadjuvant chemotherapy for women with TNBC, did not result into a significant increase of pCR rate (30.4% versus 25.9%; p 0.76), even if a downregulation of the mTOR pathway at 48 hours in the everolimus arm was observed [48]. Regarding the HR-positive/HER2-negative disease in the neoadjuvant setting, there are no studies combining chemotherapy and inhibitors of the PI3K pathway. A phase II randomized trial was carried out to evaluate the rate of tumor response combining everolimus and letrozole in the neoadjuvant treatment of postmenopausal women with HR-positive BC. The response rates were 68.1% in the everolimus arm and 59.1% in the placebo arm (p 0.062), a difference considered statistically significant in the one-sided χ2 test [49].

With respect to prognosis, our findings allow us to conclude that: a) patients with HER2-positive tumors tend to have a better long-term outcome in case of *PIK3CA* wild-type; b) patients with TNBC tend to have a better outcome in case of *PIK3CA* mutated; c) patients with HR-positive/HER2-negative tumors harboring *PIK3CA* mutation could probably still benefit from neoadjuvant chemotherapy since, although not achieving pCR, their median DFS has not yet been reached, compared to 52 months of patients with HR-positive/HER2-negative*/PIK3CA* wild-type tumors. In the HER2-positive subgroup, the overall median DFS was not still reached for patients with *PIK3CA* wild-type tumors, while it was 23 months for the *PIK3CA* mutated ones. The difference was not statistically significant (p 0.337), but a trend toward worse prognosis in case of mutation can be observed in the Kaplan–Meier curve (Figure 2). The prognostic effect of *PIK3CA* mutation within HER2-positive tumors is well established in the metastatic setting: in the CLEOPATRA trial, median PFS was longer for patients with wild-type *PIK3CA* in both the control and pertuzumab arms (8.6 months for mutated versus 13.8 months for wild-type in the control arm; 12.5 months for mutated versus 21.8 months for wild-type in the pertuzumab arm) [20]. On the other hand, in the neoadjuvant setting, only a trend toward an inferior OS for patients with a *PIK3CA* mutated tumor was reported in the Gepar experience [18], but data on survival from the pooled analysis by Loibl et al [44] could not allow to draw definite conclusions. At a median follow-up of 47 months, there was no statistically significant difference in DFS between patients with wild-type and mutated tumors (hazard ratio mutated versus wild-type 1.07; p 0.691). However, a significant interaction between HR status and *PIK3CA* genotype was described. The HR-positive subgroup showed a statistically significant longer DFS for the *PIK3CA* wild-type cohort (p 0.050), while in the HR-negative subgroup there was a non-significant trend for a longer DFS for the *PIK3CA* mutated cohort (p 0.170). This interaction cannot be confirmed in our results, as we observed a better DFS for patients with wild-type tumors both in the HR-positive and in the HR-negative subgroups compared to mutated (16 months versus not reached for HR-positive tumors, respectively; 18.5 months versus not reached for HR-negative tumors, respectively). In the triple-negative subgroup, *PIK3CA* mutations were associated with a worse long-term outcome: even if the difference was not statistically significant (p 0.115), the overall median DFS was 3 months for patients with *PIK3CA* wild-type tumors and 26 months for the *PIK3CA* mutated ones. The presence of *PIK3CA* mutations on cell-free DNA in plasma of patients with early-stage TNBC resulted as a positive prognostic factor on both RFS and BC-specific-survival [50]. A trend toward better OS for patients with TNBC harboring mutations in the PI3K pathway was also observed in a cohort of 104 TNBC cases studied by whole exome sequencing in order to decipher the genetic landscape of these tumors and identify specific mutational patterns with prognostic potential [51]. In the HR-positive/HER2-negative subgroup, patients with *PIK3CA* mutated tumors seem to have a longer DFS (not yet reached) compared to wild-type. Any patients in this subgroup achieved the pCR, but it could be hypothesized that, beside the clinicopathological parameters mentioned above (nodal status, PgR expression, Ki67) also *PIK3CA* mutation status can be considered a useful variable to indicate a chemotherapy to affected patients. Larger studies may help answer this question.

The present study has some substantial limitations, mainly related to the small number of patients in each subgroup, affecting the power of statistical analysis and results. Anyway, the strength of this work lies in the analysis of a homogeneous series of patients, all treated with the same chemotherapy regimen, and in the strictness of the *PIK3CA* mutational analysis, making it an interesting and well-done example of translational research in real-life clinical practice.

## MATERIALS AND METHODS

### Inclusion criteria and patients’ evaluation

Women with invasive, previously untreated, clinical stage II/III or inflammatory BC, histologically confirmed from a core biopsy specimen, aging 18-75, World Health Organization performance status ≤2, adequate cardiac (echocardiographic LVEF ≥50%), haematological, hepatic and renal functions were enrolled. Exclusion criteria included uncontrolled severe comorbidities, particularly ischemic heart disease (myocardial infarction, angina pectoris), arrhythmia, New York Heart Association (NYHA) grade ≥ II or congestive heart failure (CHF).

BC patients were treated in clinical practice, based on the indication of epirubicin, cyclophosphamide, docetaxel and trastuzumab approved by Agenzia Italiana del Farmaco (AIFA) for administration *in label* in Italian public hospitals and on the evaluation of the Institutional Review Board of San Salvatore Hospital, L’Aquila, Italy. All patients gave written informed consent to participate. The protocol was conducted in accordance with the Declaration of Helsinki of the World Medical Association and Good Clinical Practice.

Before every treatment cycle administration, patients underwent clinical examination and assessment of blood chemistry, toxic effects and tumor size. Toxicity was evaluated according to National Cancer Institute Common Toxicity Criteria (NCI-CTC, version 4.02). Cardiac monitoring was conducted by ECG and biochemical monitoring (blood dosage of pBNP and troponin I) at d1 of every cycle and Echocardiogram with LVEF evaluation at baseline, after the EC phase and at treatment completion.

### Study design and treatment plan

This was a single-arm, single-centre, phase I-II study of dose-dense (every 2 weeks) docetaxel (D) (starting dose 65 mg/m^2^, that is the RD reached in combination with non-pegylated liposomal doxorubicin in a dose-dense schedule, as we recently reported [52]), combined with trastuzumab (T) (4 mg/kg, loading dose 6 mg/kg) in HER2-positive patients, for 4 cycles following dose-dense epirubicin plus cyclophosphamide (EC) (90/600 mg/m^2^) for 4 cycles, as neoadjuvant chemotherapy in patients with BC. An inter-patient dose-de-escalation approach was planned, scheduling dose reduction to 60, 55 and 50 mg/m^2^ [53]. Trastuzumab dose was adapted to the biweekly administration of chemotherapy, based on the demonstrated schedule independence of its linear pharmacokinetics [54], to maintain a dose intensity of 2 mg/kg/w. Pegylated G-CSF was scheduled 24 hours after chemotherapy administration.

Dose-limiting toxicities (DLTs) were defined as G4 haematological; G3 non-haematological; any toxicity resulting in >2 weeks chemotherapy delay; ≥10% LVEF reduction from baseline, if final value was <50% or ≥20% LVEF reduction from baseline if final value was ≥50%, arrhythmia, symptomatic heart failure. Definitive surgery, either breast conservation or mastectomy with complete axillary lymph node dissection, was performed at treatment completion and recommended three weeks after chemotherapy discontinuation.

Primary end-points were: determination of D RD, in combination with T in HER2-positive patients, in a dose-dense schedule following dose-dense EC combination (phase I); the activity (measured by the pCR rate, defined as absence of invasive residual BC in the breast and axillary lymph nodes in all surgically excised specimens) and efficacy (measured by RFS, DFS and OS) (phase II) of the proposed regimen. Secondary end points included: the assessment of the correlation between activity and efficacy of treatment with the *PIK3CA* mutation status before chemotherapy, safety and compliance.

### Immunohistochemical and pathologic assessment

ER, PgR and HER2 analysis was performed on pretreatment core needle biopsy specimens using immunohistochemical staining techniques. ER and PgR positivity was defined as staining in ≥10% of cells. HER2 analysis was done using the HercepTest (Dako), with staining intensity score evaluated from 0 to 3+. For specimens staining 2+, Fluorescent *in situ* Hybridization analysis was performed to assess HER2 amplification (ratio >2.2).

To overcome the simplistic dichotomization of response in terms of pCR or residual disease, the latter including a broad range of responses, the RCB was measured for non-pCR tumors, calculated as a continuous variable derived from the primary tumor dimensions, cellularity of the tumor bed and axillary nodal burden [55].

### *PIK3CA* mutation assessment

Contiguous sections 3μm thick from the interior of a formalin-fixed paraffin-embedded (FFPE) tissue block were cut. Hematoxilyn and eosin staining was performed to assess the presence and amount of tumor cells. Genomic DNA was extracted (FFPE DNA purification kit, Norgen Biotek Corp.), by following the manufacturer's instructions. To detect hot-spot *PI3KCA* mutations, polymerase chain reaction (40 cycles) was performed by using 50ng genomic DNA, dNTPs 250μM, MgCl_2_ 1.5mM, Taq DNA polymerase (Invitrogen) 5U, primers 10pmol each (as reported by Sueta et al [56]). Amplified products were purified (Qiaquick PCR purification Kit, Qiagen). Thirty nanograms of purified PCR products were subjected to sequencing by using the Big Dye terminator v3.1 sequencing kit (Applied Biosystems) according to the manufacturer's instructions and run on a 3500 Genetic Analyzer (Applied Biosystems).

### Statistical analysis

The sample size was calculated according to the Simon's two-stage phase II optimal design. For the HER2-positive and triple-negative tumors, the null response rate (*p_0_*) below which there would be no further interest in the proposed regimen was set at 20%, and the rate beyond which further studies would be of interest (*p_1_*) was set at 40%. Assuming an α error rate of 0.05 and a β error rate of 0.20, 13 patients were to be accrued in the first step. If ≥3 pCRs were observed, 30 more patients were to be enrolled. The regimen was considered of clinical interest if ≥12 pCRs were observed among 43 patients. For the HR-positive/HER2-negative tumors, *p_0_* was set at 10% and *p_1_* was set at 30%. Assuming an α error rate of 0.05 and a β error rate of 0.20, 10 patients were to be accrued in the first step. If ≥1 pCRs were observed, 19 more patients were to be enrolled. The regimen was considered of clinical interest if ≥5 pCRs were observed among 29 patients. RFS, DFS OS were evaluated by Kaplan–Meier method [57]. RFS was evaluated from the beginning of treatment to disease recurrence or last contact; DFS from surgery to disease recurrence or last contact; OS from the beginning of treatment to death or last contact. The significance of differences between survival rates was evaluated with the log-rank test. Relationships between *PIK3CA* mutation status and clinical, histological and biological parameters were estimated with the chi-squared test.

## SUPPLEMENTARY MATERIALS TABLES



## Abbreviations

PI3K: phosphatidylinositol-3-kinase
mTOR: mammalian target of rapamycin
BC: breast cancer
HER2: human epidermal growth factor receptor 2
ER: estrogen receptor
pCR: pathological complete response
DFS: disease-free survival
OS: overall survival
HR: hormone receptor
dd: dose-dense
RFS: relapse-free survival
T: trastuzumab
RD: recommended dose
D: Docetaxel
EC: Epirubicin/Cyclophosphamide
LABC: locally advanced BC
IBC: inflammatory breast cancer
PgR: Progesterone Receptor
DLT: Dose-limiting toxicity
HFS: hand-foot syndrome
nab: nanoparticle-albumin-bound
DI: dose intensity
ITT: intention-to-treat
TNBC: triple negative breast cancer
pBNP: precursor-Brain-Natriuretic-Peptide
LVEF: left ventricular ejection fraction
RCB: Residual Cancer Burden
CHF: congestive heart failure
FFPE: formalin-fixed paraffin-embedded
OR: odds ratio
CI: Confidence Interval
AR: androgen receptor
