# Supplementary material for: Neoadjuvant chemotherapy in breast cancer: a dose-dense schedule in real life and putative role of PIK3CA mutations
Source: Oncotarget. 2018 Jun 8;9(44):27380–96. doi: 10.18632/oncotarget.25270 (PMC6007957; doi:10.18632/oncotarget.25270)
Supplement: Supplementary file 1 [file oncotarget-09-27380-s001.pdf]

Neoadjuvant chemotherapy in breast cancer: a dose-dense schedule in real life and putative role of *PIK3CA* mutations

SUPPLEMENTARY MATERIALS

Supplementary Table 1: Toxicity during the Epirubin/Cyclophosphamide phase

Epirubicin-Cyclophosphamide								
**Number**	**Patients**				**Cycles**			
	**42**				**168**			
Grade	**1**	**2**	**3**	**4**	**1**	**2**	**3**	**4**
Nausea (%)	27 (64)	6 (14)	-	-	64 (38)	7 (4)	-	-
Vomiting (%)	6 (14)	1 (2)	1 (2)	-	7 (4)	1 (0.5)	1 (0.5)	-
Diarrhoea (%)	3 (7)	2 (5)	-	-	5 (3)	2 (1)	-	-
Constipation (%)	15 (36)	3 (7)	-	-	22 (13)	4 (2)	-	-
Mucositis (%)	20 (48)	2 (5)	-	-	34 (20)	2 (1)	-	-
Asthenia (%)	19 (45)	11 (26)	3 (7)	-	59 (35)	19 (11)	6 (4)	-
HFS (%)	4 (10)	-	-	-	5 (3)	-	-	-
Neurology (%)	1 (2)	-	-	-	2 (1)	-	-	-
Myalgia (%)	4 (10)	-	-	-	6 (4)	-	-	-
Transaminase ↑ (%)	4 (10)	2 (5)	1 (2)	-	11 (7)	3 (1.7)	1 (0.5)	-
Anemia (%)	14 (33)	3 (7)	-	-	21 (12)	4 (2)	-	-
Leucopenia (%)	2 (5)	7 (17)	10 (24)	4 (10)	2 (1)	12 (7)	17 (10)	6 (4)
Neutropenia (%)	2 (5)	8 (19)	6 (14)	11 (26)	3 (1.7)	12 (7)	12 (7)	13 (8)
Thrombocytopenia (%)	2 (5)	-	-	-	3 (1.7)	-	-	-

Supplementary Table 2: Toxicity during the Docetaxel ± Trastuzumab phase

Number	Docetaxel															
	HER2-positive population (with Trastuzumab)								Her2-negative population (alone)							
	Patients				Cycles				Patients				Cycles			
	22				128				18				99			
Grade	**1**	**2**	**3**	**4**	**1**	**2**	**3**	**4**	**1**	**2**	**3**	**4**	**1**	**2**	**3**	**4**
Nausea (%)	8 (36)	-	-	-	8 (6)	-	-	-	2 (11)	1 (6)	-	-	3 (3)	1 (1)	-	-
Vomiting (%)	2 (9)	-	-	-	2 (1.5)	-	-	-	-	-	-	-	-	-	-	-
Diarrhoea (%)	3 (14)	4 (18)	-	-	3 (2)	5 (4)	-	-	2 (11)	-	-	-	2 (2)	-	-	-
Constipation (%)	6 (27)	-	-	-	9 (7)	-	-	-	4 (22)	-	-	-	6 (6)	-	-	-
Mucositis (%)	12 (55)	2 (9)	-	-	35 (27)	2 (1.5)	-	-	7 (39)	1 (6)	-	-	17 (17)	2 (2)	-	-
Asthenia (%)	7 (32)	5 (23)	4 (18)	-	43 (34)	12 (9)	4 (3)	-	5 (28)	10 (56)	2 (11)	-	42 (42)	22 (22)	2 (2)	-
HFS (%)	9 (41)	-	-	-	19 (15)	-	-	-	8 (44)	2 (11)	-	-	22 (22)	2 (2)	-	-
Neurology (%)	4 (18)	-	-	-	4 (3)	-	-	-	3 (17)	-	-	-	5 (5)	-	-	-
Myalgia (%)	15 (68)	1 (5)	-	-	34 (27)	1 (0.7)	-	-	8 (44)	3 (17)	1 (6)	-	27 (27)	4 (4)	1 (1)	-
Transaminase ↑ (%)	2 (9)	2 (9)	1 (5)	-	6 (5)	4 (3)	1 (0.7)	-	2 (11)	2 (11)	-	-	4 (4)	2 (2)	-	-
Anemia (%)	10 (45)	1 (5)	-	-	30 (23)	1 (1)	-	-	7 (39)	1 (6)	-	-	24 (24)	1 (1)	-	-
Leucopenia (%)	-	-	-	-	-	-	-	-	1 (6)	-	2 (11)	-	1 (1)	2 (2)	2 (2)	-
Neutropenia (%)	-	-	-	-	-	-	-	-	1 (6)	-	1 (6)	1 (6)	1 (1)	1 (1)	2 (2)	1 (1)
Thrombocytopenia (%)	-	-	-	-	-	-	-	-	-	-	-	-	-	-	-	-

Supplementary Table 3a: Cardiac toxicity during the Epirubin/Cyclophosphamide phase

		Epirubicin-Cyclophosphamide							
**Number**		**Patients**				**Cycles**			
		**42**				**168**			
Grade		**1**	**2**	**3**	**4**	**1**	**2**	**3**	**4**
**Cardiac Arrhythmia**	Conduction abnormality (%)	9 (21)	-	-	-	11 (7)	-	-	-
	Prolonged QTc interval (%)	-	-	-	-	-	-	-	-
	Supraventricular/ nodal (%)	1 (2)	-	-	-	1 (0.5)	-	-	-
	Ventricular (%)	-	-	-	-	-	-	-	-
	Other (%)	-	-	-	-	-	-	-	-
	**Total (%)**	10 (24)	-	-	-	12 (7)	-	-	-
**Cardiac General**	Hypertension (%)	1 (2)	2 (5)	-	-	2 (1)	3 (1.7)	-	-
	Pulmonary hypertension (%)	-	-	-	-	-	-	-	-
	Valvular heart disease (%)	9 (21)	-	-	-	9 (5)	-	-	-
	Diastolic dysfunction (%)	3 (7)	-	-	-	3 (1.7)	-	-	-
	Sistolic LV dysfunction (%)	-	-	-	-	-	-	-	-
	Other (%)	1 (2)	1 (2)	-	-	1 (0.5)	1 (0.5)	-	-
	**Total (%)**	11* (26)	3 (7)	-	-	12^ (7)	4 (2)	-	-

* Two patients reported more than one category of G1 cardiac general toxicity.

^ Two patients reported more than one category of G1 cardiac general toxicity in the same treatment cycle.

Supplementary Table 3b: Cardiac toxicity during the Docetaxel ± Trastuzumab phase

		Docetaxel-Trastuzumab (HER2-positive)								Docetaxel alone (HER2-negative)							
**Number**		**Patients**				**Cycles**				**Patients**				**Cycles**			
		**22**				**128**				**18**				**99**			
Grade		**1**	**2**	**3**	**4**	**1**	**2**	**3**	**4**	**1**	**2**	**3**	**4**	**1**	**2**	**3**	**4**
**Cardiac Arrhythmia**	Conduction abnormality (%)	6 (27)	-	-	-	12 (9)	-	-	-	3 (17)	-	-	-	5 (5)	-	-	-
	Prolonged QTc interval (%)	-	-	-	-	-	-	-	-	-	-	-	-	-	-	-	-
	Supraventricular/ nodal (%)	5 (23)	-	-	-	7 (5)	-	-	-	-	-	-	-	-	-	-	-
	Ventricular (%)	-	-	-	-	-	-	-	-	-	-	-	-	-	-	-	-
	Other (%)	-	-	-	-	-	-	-	-	-	-	-	-	-	-	-	-
	**Total (%)**	9* (41)	-	-	-	18♦ (14)	-	-	-	3 (17)	-	-	-	5 (5)	-	-	-
**Cardiac General**	Hypertension (%)	1 (5)	2 (9)	-	-	3 (2)	3 (2)	-	-	1 (6)	-	-	-	2 (2)	-	-	-
	Pulmonary hypertension (%)	-	-	-	-	-	-	-	-	-	-	-	-	-	-	-	-
	Valvular heart disease (%)	3 (14)	-	-	-	3 (2)	-	-	-	1 (6)	-	-	-	1 (1)	-	-	-
	Diastolic dysfunction (%)	2 (9)	-	-	-	2 (1.5)	-	-	-	1 (6)	-	-	-	1 (1)	-	-	-
	Sistolic LV dysfunction (%)	-	-	-	-	-	-	-	-	-	-	-	-	-	-	-	-
	Other (%)	-	2 (9)	-	-	-	-	-	-	1 (6)	-	-	-	1 (1)	-	-	-
	**Total (%)**	4^ (18)	2° (9)	-	-	7† (5)	3 (2)	-	-	3^ (17)	-	-	-	4 (4)	-	-	-

* Two patients reported more than one category of G1 cardiac arrhythmia.

♦ One patient reported more than one category of G1 cardiac arrhythmia in the same treatment cycle.

^ One patient reported more than one category of G1 cardiac general toxicity.

° Two patients reported more than one category of G2 cardiac general toxicity.

† One patient reported more than one category of G1 cardiac general toxicity in the same treatment cycle.
